# The neuropsychological profile of work addiction

**DOI:** 10.1038/s41598-023-47515-9

**Published:** 2023-11-16

**Authors:** Krisztina Berta, Zsuzsanna Viktória Pesthy, Teodóra Vékony, Bence C. Farkas, Dezső Németh, Bernadette Kun

**Affiliations:** 1 Institute of Psychology, ELTE Eötvös Loránd University, Budapest, Hungary; 2 Doctoral School of Psychology, ELTE Eötvös Loránd University, Budapest, Hungary; 3 Centre de Recherche en Neurosciences de Lyon CRNL U1028 UMR5292, INSERM, CNRS, Université Claude Bernard Lyon 1, Bron, France; 4 Université Paris-Saclay, UVSQ, INSERM, CESP, Villejuif, France; 5 Institut du Psychotraumatisme de l’Enfant et de l’Adolescent, Conseil Départemental Yvelines et Hauts-de-Seine et Centre Hospitalier des Versailles, Versailles, France; 6 Centre de Recherche en Épidémiologie et en Santé des Populations, INSERM U1018, Université Paris-Saclay, Université Versailles Saint-Quentin, Paris, France; 7 BML-NAP Research Group, Institute of Psychology, Institute of Cognitive Neuroscience and Psychology, HUN-REN Research Centre for Natural Sciences, Eötvös Loránd University, Budapest, Hungary

**Keywords:** Neuroscience, Psychology

## Abstract

The objective of this study was to examine, for the first time, the neuropsychological aspects of work addiction, with a specific emphasis on the cognitive factors identified by theoretical models. While previous research has highlighted self-reported obsessiveness and impulsiveness in work addiction, this study sought to go beyond self-report measures by employing also neuropsychological reaction time tasks to assess executive functions. A total of 101 participants were categorized into two groups based on their Work Addiction Risk Test scores: a high-risk group (HWA; n = 39) and a low-risk group (LWA; n = 62) for work addiction. Executive functions were assessed using Go/No-Go, Digit Span, Counting Span, N-back, and Card Sorting Tasks. The findings revealed that the HWA group had poorer inhibitory control and achieved lower scores on the more complex working memory task involving updating (2-back). However, they exhibited unaltered cognitive flexibility and outperformed the LWA group on the 1-back task associated with maintenance and storage of information and sustained attention. Higher levels of impulsiveness and compulsiveness were observed in the HWA group, consistent with previous studies. These findings highlight the role of inhibition and working memory in work addiction, potentially contributing to challenges such as inefficient working strategies and impaired social functioning. This study offers valuable insights into the neurocognitive aspects of work addiction, deepening our understanding of this phenomenon.

## Introduction

In recent years, there has been increasing focus and interest in gaining a deeper understanding of the nature of work addiction. Over the years, empirical research has emphasized the negative consequences and symptoms of this behavior addiction as it can potentially cause impairments in one’s health and social relationships. Although work addiction shares similarities with other addictive disorders on psychological and physical levels, it is not included in the DSM-5^1^ or ICD-11^2^ as a mental disorder. Representative studies show that it affects a larger share of the population, as its prevalence varies between 7.3 and 39.7% according to specific countries^3–5^. The concept of “workaholism” first appeared in in Oates’ book^6^, defining "workaholic'' as a “person whose need for work has become so excessive that it creates noticeable disturbance or interference with his bodily health, personal happiness, interpersonal relations, and with his smooth social functioning”. Since then, there is an ongoing debate about this behavioral addiction as it is surrounded by many conflicting opinions, beliefs, and definitions^7^. While a generally accepted set of criteria or definition remains elusive, work addiction can be conceptually framed using the "components model", which elucidates its characteristics through the six core components common to other addictive disorders: salience, mood modification, tolerance, withdrawal symptoms, conflict, and relapse^8,9^. This means that in work addiction, work becomes so central to a person's life that everything else takes a back seat. They use work to manage their emotional states, work increasingly to achieve the same positive effect, experience psychological withdrawal symptoms when prevented from working, encounter intrapsychic and intrapersonal conflict due to overwork, and revert to their original workload when attempting to reduce it.

Despite the increasing amount of research on the topic of work addiction, to the best of our knowledge, the underlying possible cognitive mechanisms of this behavioral addiction have never been investigated. The objective of the current investigation is to expand the existing knowledge on the underlying factors of work addiction. Specifically, this study intends to elucidate the neurocognitive basis of work addiction by exploring the cognitive functions of cognitive flexibility, inhibitory control, and working memory.

### Executive functions and behavioral addictions

Empirical studies suggest the importance of altered executive functioning in the development and maintenance of different addictive disorders^10^. While there are various models of executive functions, many of them emphasize three core aspects. In Miyake and colleagues’^11^ framework, these functions are referred to as inhibition, shifting, and updating. Diamond^12^ also adopted the same model for the concept of executive functions but labeled them as inhibitory control, cognitive flexibility, and working memory. These terms are widely used in the literature^13–15^. To ensure consistency and comparability, we will adopt this model in our theoretical framework. These executive functions involve higher-level cognitive processes essential for goal-directed behavior, such as reduced ability to control behavior and a strong urge to use substances; it is understandable why their alterations might be associated with addictive disorders^16,17^. As this aspect of work addiction has not been investigated before, it is an important starting point to see how other behavioral addictions are related to executive functions.

While a large body of empirical research focused on the cognitive mechanisms of substance use disorders, there have been limited studies exploring the relationship between behavioral addictions and executive functions. Mostly four behavioral addictions have been investigated in this regard: gambling disorder, internet gaming disorder, compulsive buying disorder, and problematic smartphone use. Various studies have indicated that groups with these behavior addictions often performed significantly worse on tasks measuring inhibitory control^18–25^, cognitive flexibility^20,22,25–28^ and working memory^19,24,25,27,29,30^ compared to healthy controls. It is important to note that a part of these studies showed contradictory results as in some cases groups with behavioral addictions showed intact or selectively affected executive functioning^24,25,28,30–32^.

Taken together, these findings suggest that there is a possible selective alteration of executive functions not only in substance use disorders but behavioral addictions as well, as in several executive function tasks, the groups with behavioral addictions showed impaired executive functioning compared to controls. Since work addiction is similar in many aspects to other behavioral addictions, the question may arise whether this can be assumed to be the case for executive functioning as well.

### Characteristics of work addiction

Not only the similarities with other behavioral addictions, but also certain characteristics could indicate altered executive functions in work addiction. A great example of this is that workaholics often have difficulty disengaging from work processes, they tend to persevere in their own work and continue to work, despite not enjoying it^33^. Several theories emphasize that work addiction encompasses not only behavioral symptoms, such as overcommitment and work-life conflict, but also a cognitive component^34,35^. Workaholics struggle with mentally disengaging from their tasks, experiencing compulsive preoccupation with work, and feeling an inner compulsion to continuously engage in work. Studies also suggest that these people find it more difficult to delegate tasks, and have a greater need to maintain control^36–38^. A growing evidence suggests an association between work addiction and impulsiveness, compulsiveness, and obsessive–compulsive symptoms^36,39–41^. Despite the positive correlations between work addiction and these characteristics, very little focus has been directed towards a deeper understanding of the possible neurocognitive background of these relationships. Given the potential link between executive functions and various addictions^8^, it appears important to explore the neuropsychological aspects possibly associated with work addiction. Our study is intended to address potential gaps in this area.

The present study aimed to compare executive functions, namely inhibitory control, cognitive flexibility, and working memory in individuals with high-risk (HWA) versus low-risk of work addiction (LWA). Moreover, we aimed to compare these groups on specific personality variables that are closely related to cognitive functions. Based on previous studies, we expected that the HWA group would perform significantly worse on the tasks measuring executive functions and would show significantly higher obsessive-compulsiveness and impulsiveness.

## Methods

### Participants

A total of 111 participants from Hungary took part in the study. The inclusion criteria were the absence of any neurological and psychiatric disorder, having an active employment, and being at least 18 years old. Ten participants were excluded because of self-reported current psychiatric disorder. Thus, a total of 101 participants' data were analyzed (*M*_age_ = 41.10 years; *SD* = 9.29, 39.22% males). Notably, this sample size is either comparable to or larger than those reported in most similar studies within the field^20,22,27,29^.We divided them into two groups: a high risk for work addiction group (HWA, *n* = 39; *M*_age_ = 37.51 years, *SD* = 7.81 years; 30.8% males) and a low risk for work addiction group (LWA, *n* = 62; *M*_age_ = 42.8 years, *SD* = 9.96 years; 56% male), based on their scores on the Work Addiction Risk Test (WART)^42^. In addition, for some tasks, data from additional participants had to be excluded due to assessment issues (e.g., failure of saving data, misunderstanding the instructions of the tasks, lack of motivation) and we also excluded those who scored outside two standard deviations (see Supplementary Materials S1.1). The database can be found with and without excluding outliers on OSF (see Data availability statement). Regarding their educational level, the majority (85.29%) of the participants had a college or university diploma, five participant (4.9%) had a doctoral degree, nine of them (8.2%) had a high school diploma, and one participant (0.09%) had a vocational training without a high school diploma. Around three quarters (73.55%) of the participants lived in the capital city, 20 of them (19.61%) in another city/town, and six of them (4.9%) in a village or hamlet.

### Measures

#### Tasks measuring executive functions

JavasScript jsPsych library^43^ were used to create and run a computerized version of the following cognitive tasks. The source codes of the tasks are openly available on the link provided in the Data Availability Statement.

##### Cognitive flexibility

Cognitive flexibility was measured by Card Sorting Task (CST^44,45^ based on the concept of the Wisconsin Card Sorting Test (WCST^46^ that assesses executive functions such as cognitive flexibility^47^. In this task, four cards appear on the screen and various items are shown on each card. The items may be in different colors (red, yellow, green, or blue), numbers (1–4 items), and shapes (triangle, star, diamond, or circle). At the same time, another card is displayed below the four cards and the participant is instructed to classify this card to one of the top four cards based on color, number of items, or shape by clicking on the selected card. After each response, feedback is given to the participant indicating whether the response was correct. The rule is not provided to the participants; they have to rely on the feedback to figure out the correct rule. A total of 64 cards are presented to the participant and the categorization rule changes after ten correct answers in a row. The rule changes in the following order: color, shape, number, color, shape, and number. Cognitive flexibility is measured by the number of perseverative errors, i.e., where the participant makes an error using the same rule as for the previous answer. Less perseverative error means better cognitive flexibility.

##### Inhibitory control

Go/No-Go tasks are widely used measures of inhibitory control^48^. We used a modified version of the Go/No-Go task^49,50^. In this version of the Go/No-Go task, a 2 × 2 square grid is presented on the screen with one blue star in each of them. A letter P or R appears randomly in place of one of the stars. The participants are asked to press the spacebar when the letter P (Go trials) appears, and not to press it when the letter R (No-Go trials) appears. Halfway through the task, the instructions change: if the letter R appears, press the spacebar, if the letter P appears, press nothing. The task begins with a practice block of 20-trials, when the participant receives feedback on the correctness of their response Then, two blocks of 160–160 stimuli follow each other (no feedback is provided). The ratio of the Go and No-Go trials is 80:20. The letters are displayed until a correct response is given (with a maximum response time of 500 ms). Trials follow each other with 1500 ms response-to-stimulus interval. Performance on the task was assessed by three scores: correct response to the Go trials (hit), incorrectly pressing the spacebar to the No-Go trials (false alarm) and a standardized value of the hit to false alarm ratio (d-prime), which scores are often used to measure inhibition^51^, with higher scores indicating greater discriminability. D-prime, hereinafter referred to as the discriminability score, was computed individually as follows:

$$ Discriminability \, score \, (d - prime) \, = \, Z\left( {hit \, rate} \right) \, - \, Z \, \left( {false \, alarm \, rate} \right) $$

##### Working memory

We used three tasks to assess working memory: Digit Span, Counting Span, and N-back tasks. All of them are commonly used to measure working memory but show weak correlations with each other^52^. Thus, applying all these tasks, we could investigate several aspects of working memory. The Counting Span and N-back tasks were adapted with the jsPsych library by Vékony^53^.

The Counting Span Task^54^ (CSPAN) measures complex working memory. In this task, circles and squares are presented on the screen, colored yellow or blue. Participants are instructed to count the blue circles out loud, repeat the result of the counting, and memorize this number. Further trials are then followed with the same task. After a few trials, a question mark appears on the screen and the participant has to recall the memorized numbers in the correct order. The task starts with two trials in a block (e.g., the question mark appears after two trials, and the participant has to retrieve the two memorized numbers) and then the number of trials increases within a block, therefore, participant has to keep more and more digits in mind while counting the blue circles out loud. If they cannot recall the final numbers correctly in order, the investigator starts a new session. The task consists of practice sessions with three blocks (with 2–2 trials/block) and three actual sessions. The average of the three sessions gives the counting span score of a participant.

The N-Back Tasks^55^ are also widely used to measure working memory. In this task, letters appear on the screen (B, K, Q, T, H, M, N, P, X, or R). In the first half of the task (1-back), participants are asked to decide whether the letter that is currently on the screen (n) is the same as the letter that appeared before it (n − 1). In the second part of the task (2-back), the task is to decide if the current letter that appeared two trials prior (n − 2). If it is the same letter, the participant has to press the J key on the keyboard; if not, the F key has to be pressed. The target ratio is set at 20% (20 targets, 80 non-targets at each level). The 1-back and 2-back tasks follow one after the other, each with a 10-trial practice session at the beginning with feedback on whether the answer was correct. Then, a total of 100 letters are then displayed at each level. The stimuli appear for 500 ms or until one of the response keys is pressed, with an interstimulus interval of 1500 ms. For the analysis of this task, we also used the number of correct responses, the false alarms, their reaction times, and the discriminability scores, computed similarly as for the Go/No-Go task.

We used the Digit Span Task^56,57^ (DSPAN) to measure phonological working memory capacity. The examiner reads aloud a series of digits for the participants. The participant is asked to memorize these number sequences and then recall them in the correct order. Initially, the participant has to recall a series of three digits. There are a total of four different sets of three-digit numbers. If the subject can recall at least three of these correctly, the examiner moves on to the four-digit numbers, and so on. The digit span score of a participant is the number of digits in the longest series completed.

#### Self-report questionnaires

##### Work addiction

The risk of work addiction was measured by the original, 25-item version of Work Addiction Risk Test (WART^42^, which was adapted to Hungarian by Urbán et al.^58^. The items are rated on a four-point Likert scale from ’never true’ to ’always true’. The higher the score on the scale means the higher risk of work addiction. We divided the participants into two groups based on the WART scores. In our study, we applied the empirically validated 67-point cut-off criterion, widely recognized for this instrument, as empirically established by Robinson et al.^59^ This classification resulted in individuals with a score below 67 points falling into the LWA category, while those with 67 points or higher on the WART were designated as HWA. The scale has a good internal reliability on our sample (*α* = 0.87).

##### Impulsiveness

Impulsiveness was assessed by the 21-item modified version of the Barratt Impulsiveness Scale (BIS)^60,61^. Items are rated by the participant on a 4-point Likert scale (1—never/rarely, 2—sometimes, 4—always). A higher score indicates a higher level of impulsiveness. Three factors are identified in the questionnaire: cognitive impulsivity, behavioral impulsiveness, and impatience/agitation. In the present study, we used only the total score of the BIS. The scale showed good reliability in our sample (*α* = 0.82).

##### Obsessiveness

We used the Five-Factor Obsessive–Compulsive Inventory Short Form (FFOCI)^62,63^ to assess obsessive and compulsive traits. The scale contains 48 items that have to be rated on a 5-point Likert scale (ranging from “strongly disagree” to “strongly agree”). A higher score indicates a higher level of obsessiveness. The inventory has a good internal reliability in our sample (*α* = 0.89).

### Procedure

We recruited the participants from the database of a previous study examining the relationship of work addiction and personality factors^64^. In the previous study, we utilized a snowball sampling method to gather participants for an online questionnaire survey, where they completed several questionnaires, including one assessing work addiction. At the end of the online questionnaire, participants willingly provided their contact information, indicating their interest in potentially taking part in future research. While work addiction generally displays stability^65–67^), individual cases may occasionally undergo spontaneous recovery or other changes over time. Therefore, though we invited individuals to participate based on their score of the Work Addiction Risk Test^42,58^ of this previous study, we based the final group assignments on the participants' actual WART scores from this study to accommodate potential fluctuations in work addiction levels.

At the beginning of each session, subjects were informed about the study procedure and signed an information and informed consent form. Participants took part in a two-hours long session, during which they performed various neuropsychological reaction time tasks, answered questions (regarding socio-demographic data, as well as possible influencing factors relevant to the research, e.g. illness, substance use), and filled out online questionnaires, in the same order for all participants, which was as follows: socio-demographic questions, Go/No-Go, N-back, DSPAN, CST, and CSPAN. The subjects received a gift voucher for their participation. The current study received approval from the Research Ethics Committee of the Faculty of Education and Psychology at Eötvös Loránd University (registration number 2020/401), and we followed the guidelines of the Declaration of Helsinki.

### Statistical analysis

The IBM SPSS Statistics (Version 28) and JASP (Version 0.17.2) were used for statistical analyses^68,69^ and RStudio (Version 2023.03.0) was to create the plots^70^. Given the sensitivity of cognitive functions to sociodemographic characteristics, we initially compared the HWA and LWA groups in terms of gender, age, education, and place of residence using independent sample t-test and Mann–Whitney tests depending on normality. Then, we compared performance on the neuropsychological tests and the questionnaire scores between the LWA and HWA groups. In the absence of a difference, we would employ one-way analysis of variance (ANOVA), and in the case of any significant difference, one-way analyses of covariance (ANCOVA) would be applied, with 'group' (LWA vs. HWA) as a between-subjects factor and the covariate. The homogeneity of regression slopes assumption was tested by assessing the significance of the Covariate*Group interactions terms, and it was met in each case. We test the assumptions of normality and homogeneity of variance using Shapiro–Wilk, and Levene’s tests, respectively. As with sample sizes as large as ours, ANCOVAs are robust against violations of normality and homogeneity of variance^71,72^, we report the results of standard, parametric ANCOVA in the main text. For variables violating either of these assumptions, we also report the results of robust ANCOVA, based on trimmed means^73^ in Supplementary Material S4. This method compares the dependent variable between the two groups, at specific levels of the covariate, for which the relationship between the dependent variable and the covariate is comparable across groups. These analyses were carried out in RStudio (Version 2023.03.0)^70^ using the WRS2 package^74^.

Additionally, we conducted correlational analyses between the WART scores and the results of the cognitive tasks. Normality was tested applying the Shapiro–Wilk test. When the data did not meet the assumptions of normality, Spearman's rank correlation was employed. Conversely, when the data was normally distributed, Pearson's correlation coefficient was utilized. Finally, to examine the relationships between WART total scores and our explanatory variables, we used a hierarchical multiple linear regression analysis with two blocks of predictors. In the first block, we used the following independent variables to predict WART scores: BIS total score, FFOCI total score, and age. Subsequently, in the second block, we included the following variables as additional predictors: CSPAN, DSPAN, 1-back, 2-back and Go/No-Go discriminability scores, and WCST perseverative errors. All analyses were two-tailed and were conducted with a significance level of *p* < 0.05.

## Results

When comparing the HWA and LWA groups, a significant difference was found for age: members of the LWA group were older than members of the HWA group (*t*(97) = 2.711, *p* = 0.008, *M*_LWA_ = 42.721, *SD*_LWA_ = 10.017, *M*_HWA_ = 37.526, *SD*_HWA_ = 7.918, *d′* = 0.560). However, there were no significant differences between the two groups in terms of gender, education, and place of residence (see Suppl. S1.2, Table S1). Hence, age was consistently included as a controlled variable in subsequent analyses.

**Figure 1:**
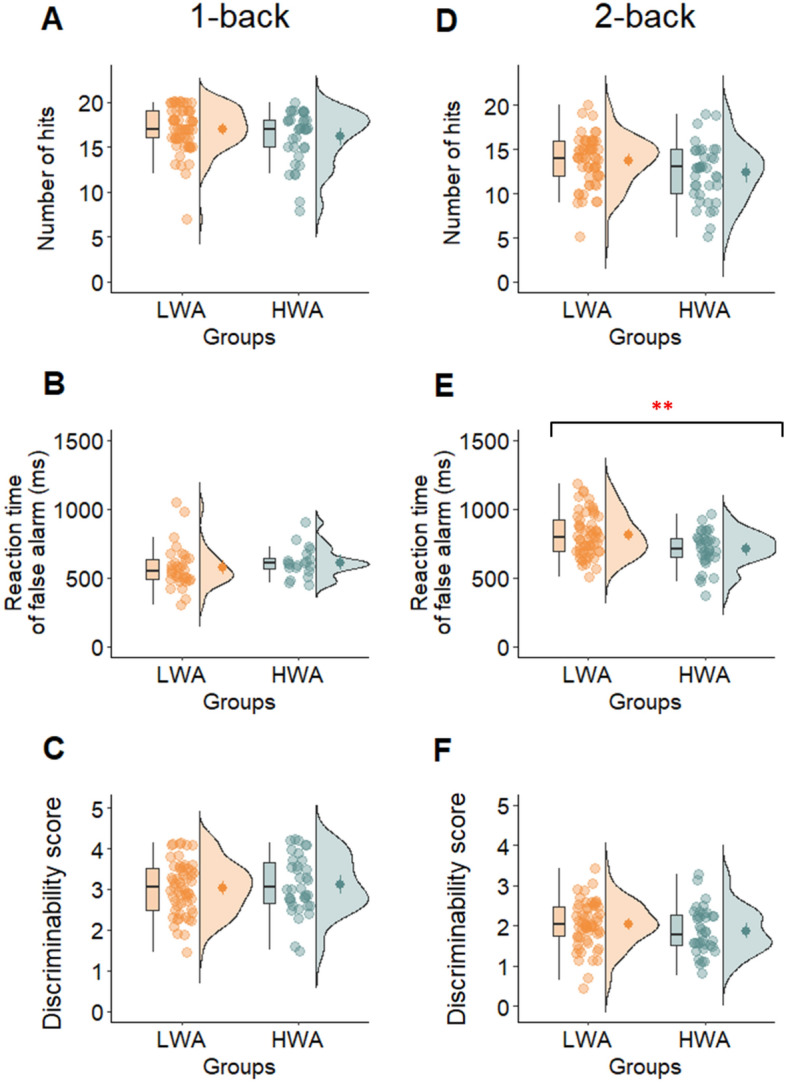
The scores of the groups low risk (LWA) and high risk (HWA) groups for work addiction on the 1-back and 2-back tests measuring working memory. In the first column, the figures show the results of the 1-back test: (**A**) the number of hits, (**B**) the reaction times of false alarms, and (**C**) the discriminability scores. The scores of the 2-back test, the (**D**) number of hits, (**B**) reaction time of false alarms, and (**D**) discriminability scores are in the second column. The red star indicates significant difference between the groups (**p < 0.01).

**Figure 2:**
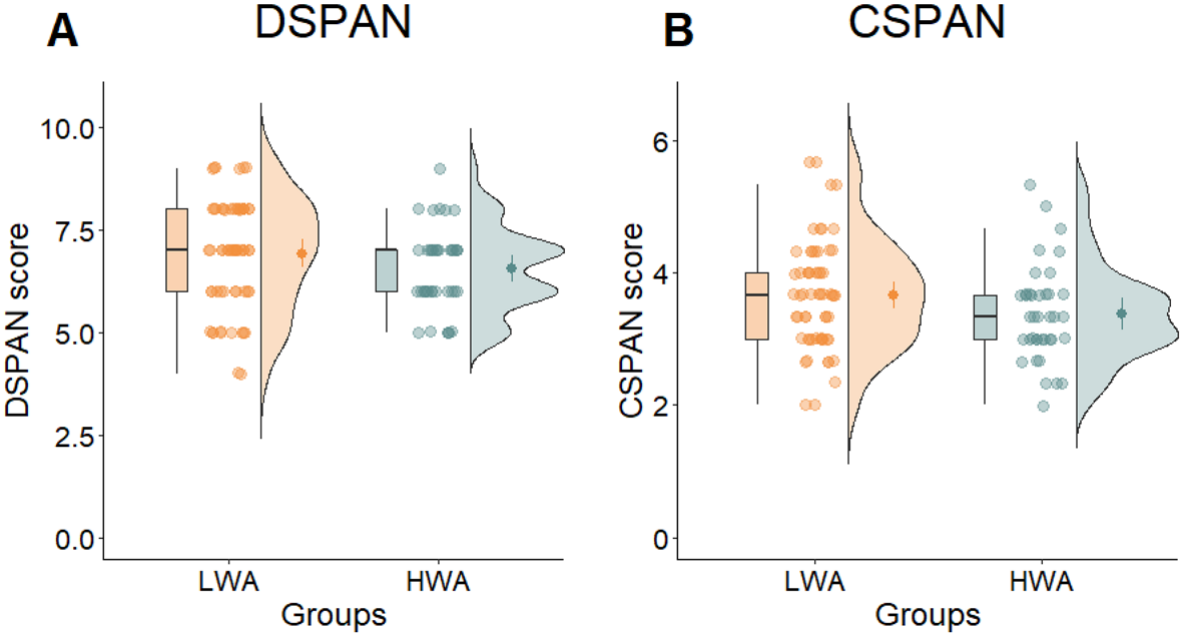
Scores of the groups measuring working memory. The (**A**) digit span (DSPAN) and (**B**) counting span (CSPAN) scores of the low risk (LWA) and high risk (HWA) groups for work addiction.

To investigate the association between work addiction on working memory performance, we compared the performance of the HWA and LWA groups on the N-back, CSPAN, and DSPAN scores, while controlling for age. We did not find a significant group difference between HWA and LWA groups on the DSPAN and CSPAN tasks and on the scores of the 1-back task after controlling for age (see Table 1 and Figs. 1 and 2). In the 2-back task, the covariate age was not significantly related to reaction time for false alarms (*F*(1, 86) = 0.101, *p* = 0.752, η^2^_p_ = 0.001). However, there was a significant effect of group after controlling for age (*F*(1, 86) = 7.960, *p* = 0.006, η^2^_p_ = 0.085). Specifically, the HWA group exhibited a faster reaction time for false alarms (*M* = 708.735, *SD* = 131.734) compared to the LWA group (*M* = 811.456, *SD* = 157.839). There was no significant group difference on the number of false alarms in this task (see Table 1). The results of robust ANCOVAs generally aligned with parametric ones, except for one instance. In the robust ANCOVA, a notable group difference surfaced for DSPAN, indicating smaller DSPAN scores in the HWA group around age 32 (p = 0.025) (see Supplementary Material S4).

**Table 1:** The results of the one-way ANCOVA comparing HWA and LWA groups.

Variable	HWA		LWA		Source	df, error df	*F*	*p*	η^2^_p_	Cohen’s d	Cohen’s d 95% CI
N (LWA, HWA)	*M*	*SD*	*M*	*SD*							
Go/No-Go 96 (37, 59)											
Number of hits	228.82	16.12	233.58	15.50	Group	1, 93	7.472	0.008	0.074	0.301	[–0.109, 0.710]
	–	–	–	–	Age	1, 93	14.553	< .001	0.135		
Number of false alarms	27.05	9.64	24.85	12.21	Group	1, 93	1.195	0.277	0.013	0.200	[–0.607, 0.208]
	–	–	–	–	Age	1, 93	1.222	0.272	0.013		
Discriminability score	1.54	0.56	1.79	0.72	Group	1, 93	7.512	0.007	0.075	0.389	[–0.023, 0.797]
	–	–	–	–	Age	1, 93	10.420	0.002	0.101		
CST	7.15	2.69	6.65	2.06	Group	1, 94	1.498	0.224	0.016	0.210	[–0.607, 0.202]
97 (38, 59)	–	–	–	–	Age	1, 94	0.942	0.334	0.010		
1-back 90 (34, 56)											
Number of hits	16.14	2.95	17.00	2.49	Group	1, 87	1.344	0.249	0.015	0.314	[–0.112, 0.737]
	–	–	–	–	Age	1, 87	0.348	0.557	0.004		
Number of false alarms	1.4	1.85	1.3	1.76	Group	1, 87	0.739	0.392	0.008	0.056	[–0.477, 0.365]
	–	–	–	–	Age	1, 87	2.291	0.134	0.026		
Reaction time of hits	546.86	70.27	562.81	89.45	Group	1, 87	0.201	0.655	0.002	0.198	[–0.224, 0.620]
	–	–	–	–	Age	1, 87	14.727	< .001	0.145		
Reaction time of false alarms	612.7	108.83	574.49	152.23	Group	1, 48	0.913	0.344	0.019	0.289	[–0.846, 0.271]
51 (19, 32)	–	–	–	–	Age	1, 48	2.742e -4	0.987	5.713e –6		
Discriminability score	3.12	0.7	3.03	0.64	Group	1, 90	–0.586	0.559	0.124	0.124	[–0.546, 0.297]
	–	–	–	–	Age	1, 90	1.955	0.166	0.022		
2-back 92 (36, 56)											
Number of hits	12.35	3.29	13.74	2.81	Group	1, 89	3.434	0.067	0.037	0.453	[0.030, 0.871]
	–	–	–	–	Age	1, 89	0.142	0.707	0.002		
Number of false alarms	5.65	3.49	6.14	3.97	Group	1, 89	1.624	0.206	0.018	0.131	[–0.283, 0.545]
	–	–	–	–	Age	1, 89	5.176	0.025	0.055		
Reaction time of hits	704.43	134.09	734.6	111.55	Group	1, 89	0.432	0.513	0.005	0.245	[0.266, 1.143]
	–	–	–	–	Age	1, 89	0.783	0.379	0.009		
Reaction time of false alarms	708.74	131.73	811.46	157.84	Group	1, 86	7.960	0.006	0.085	0.707	[–0.145, 0.687]
89 (33, 56)	–	–	–	–	Age	1, 86	0.101	0.752	0.001		
Discriminability score	1.87	0.57	2.03	0.57	Group	1, 89	0.640	0.426	0.007	0.272	[–0.172, 0.660]
	–	–	–	–	Age	1, 89	1.396	0.241	0.015		
DSPAN	6.54	1,00	6.92	1.32	Group	1, 95	2.639	0.108	0.027	0.325	[–0.081, 0.728]
98 (38, 60)	–	–	–	–	Age	1, 95	0.847	0.360	0.009		
CSPAN	3.38	0.72	3.66	0.81	Group	1, 95	2.102	0.150	0.022	0.372	[–0.035, 0.776]
98 (38, 60)	–	–	–	–	Age	1, 95	1.087	0.300	0.011		
BIS	41.46	7.85	37.00	6.32	Group	1, 96	10.707	0.001	0.100	0.626	[–1.038, –0.210]
99 (38, 61)	–	–	–	–	Age	1, 96	1.026	0.314	0.011		
FFOCI	160.54	18.98	142.66	19.67	Group	1, 96	17.475	< .001	0.154	0.925	[–1.347, –0.498]
99 (38, 61)	–	–	–	–	Age	1, 96	0.436	0.510	0.005		

*LWA* low risk of work addiction group, *HWA* high risk of work addiction group, *CST* card sorting test, *DSPAN* digit span task, *CSPAN* counting span task, *BIS* Barratt impulsiveness scale, *FFOCI* five-factor obsessive–compulsive inventory.

Weak but significant correlation was found between WART scores and two scores of the 2-back task: the number of hits and the reaction of false alarms. The correlations between the WART total scores and the other working memory scores were weak and nonsignificant, which supports the results obtained from the comparison of the working memory results between the HWA and LWA groups (Table 2).

**Table 2:** Correlations between WART score and the scores of tasks measuring executive functions, BIS, and FFOCI.

Variable	n (HWA, LWA)	WART
Go/No-Go	98 (38, 60)	
Number of hits		–0.176
Number of false alarms		0.068
Discriminability score		–0.174
CST	99	0.056
1-back		
Number of hits	92	–0.175
Number of false alarms	92	0.026
Reaction time of hits	92	0.007
Reaction time of false alarms	53	0.178
Discriminability score	92	0.098
2-back		
Number of hits	94	–0.211*
Number of false alarms	94	0.018
Reaction time of hits	94	–0.051
Reaction time of false alarms	91	–0.215*
Discriminability score	94	–0.125
DSPAN	100	–0.035
CSPAN	100	–0.087
BIS	101	0.289**
FFOCI	101	0.529***

*LWA* low risk of work addiction group, *HWA* high risk of work addiction group, *CST* card sorting test, *DSPAN* digit span task, *CSPAN* counting span task, *BIS* Barratt impulsiveness scale, *FFOCI* five-factor obsessive–compulsive inventory.

*p < 0.05; **p < 0.01; ***p < 0.001.

**Figure 3:**
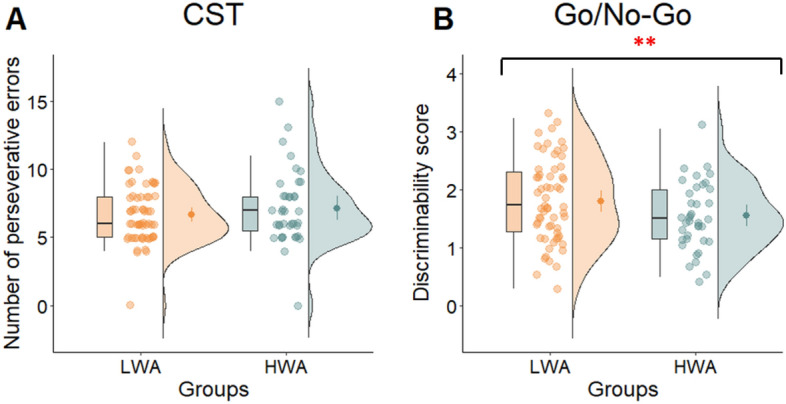
Scores of the groups measuring cognitive flexibility and inhibitory control. The (**A**) number of perseverative errors in the Card Sorting Task (CST) and (**B**) the discriminability scores of the Go/No-Go task of the low risk (LWA) and high risk (HWA) groups for work addiction (**p < 0.01).

In the CST task, the covariate age was not significantly related to the number of perseverative errors (*F*(1, 93) = 0.942, *p* = 0.334, η^2^_p_ = 0.010). There was also no significant effect of group after controlling for age (*F*(1, 93) = 1.498, *p* = 0.224, η^2^_p_ = 0.016). The mean number of perseverative errors was 6.65 (*SD* = 2.06) in the HWA group and 7.15 (*SD* = 2.69) in the LWA group (see Table 1 and Fig. 3). The correlation between the number of perseverative errors and the WART total score was not significant (*r*(*99*) = 0.056, *p* = 0.582), indicating that the degree of cognitive flexibility is not associated with a higher work addiction risk (Table 2).

The covariate age was significantly related to the Go/No-Go number of hits (*F*(1,93) = 14.553, *p* < 0.001, η^2^_p_ = 0.135). There was also a significant effect of group after controlling for age (*F*(1,93) = 7.472, *p* = 0.008, η^2^_p_ = 0.074), with a smaller number of hits in the HWA (*M* = 228.459, *SD* = 16.186), than the LWA group (*M* = 233.797, *SD* = 15.547). Similarly, in the Go/No-Go task, age was significantly related to the discriminability scores (*F*(1, 93) = 10.42, *p* = 0.002, η^2^_p_ = 0.101). After controlling for age, there was also a significant effect of the group on the discriminability scores (*F*(1, 93) = 7.51, *p* = 0.007, η^2^_p_ = 0.075). The HWA group had a lower mean discriminability score (*M* = 1.54, *SD* = 0.56) compared to the LWA group (*M* = 1.79, *SD* = 0.72). In the number of false alarms, there was no significant group difference (see Table 1 and Fig. 3).

The correlations between the WART scores and the Go/No-Go scores were not significant, indicating that there is no relationship between inhibitory control and work addiction (Table 2).

**Figure 4:**
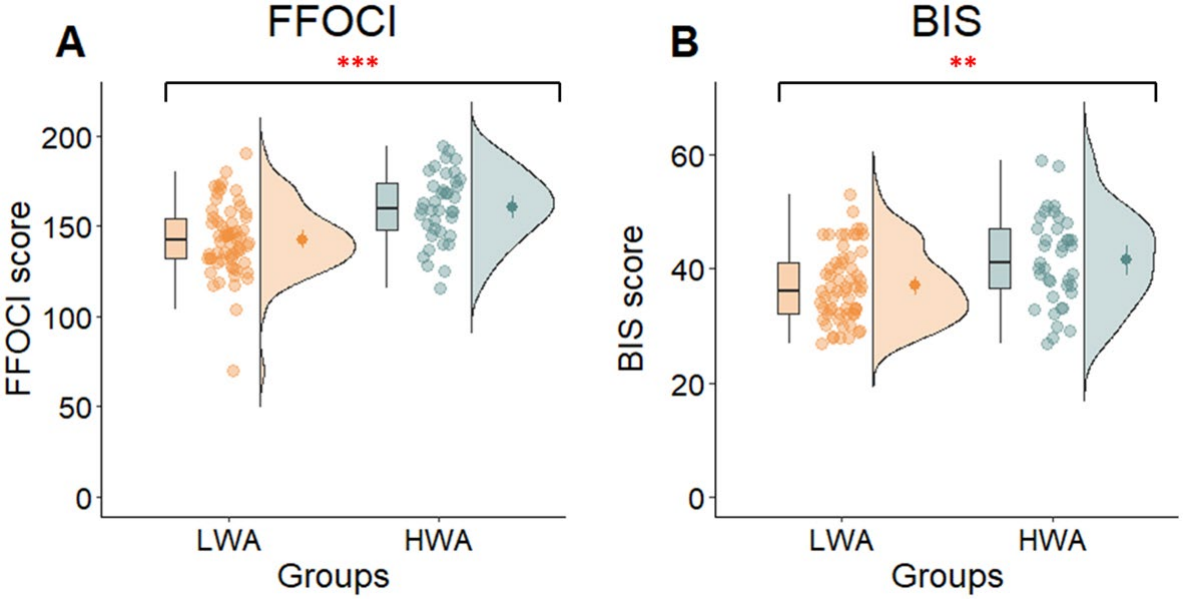
Compulsiveness and impulsiveness scores of the groups. The figure (**A**) shows the scores of the Five-Factor Obsessive–Compulsive Inventory Short Form (FFOCI), the figure (**B**) the Barratt Impulsiveness Scale (BIS) scores of the low risk (LWA) and high risk (HWA) groups for work addiction. The red star indicates significant difference between the groups (**p < 0.01; ***p < 0.001).

For the BIS measure of impulsiveness, the covariate age was not significantly related to the scores (*F*(1, 96) = 1.03, *p* = 0.31, η^2^_p_ = 0.01). However, there was a significant group difference (*F*(1, 96) = 10.71, *p* = 0.001, η^2^_p_ = 0.10). The HWA group scored significantly higher in impulsiveness (*M* = 41.46, *SD* = 7.85) compared to the LWA group (*M* = 37.00, *SD* = 6.32). Similarly, for the FFOCI, measuring compulsiveness, age was not significantly related to the scores (*F*(1, 96) = 0.44, *p* = 0.51, η^2^_p_ = 0.01). After controlling for age, there was a significant group difference (*F*(1, 96) = 17.48, *p* < 0.001, η^2^_p_ = 0.15). The HWA group had higher scores (*M* = 160.54, *SD* = 18.98) compared to the LWA group (*M* = 142.66, *SD* = 19.67) (see Fig. 4).

To further explore the relationship between the degree of work addiction and trait compulsiveness and trait impulsiveness we conducted correlation analysis. The positive correlation between the WART scores and FFOCI scores were moderate and significant, indicating that higher trait compulsiveness is associated with a higher work addiction risk. Regarding the relationship between the BIS scores and WART scores, we found a weak but significant positive correlation, indicating that higher trait impulsiveness is similarly somewhat associated with a higher work addiction risk (Table 2).

To investigate the relationships between WART total scores and our explanatory variables, we conducted a hierarchical multiple linear regression analysis with two blocks of variables. In the first step, we predicted WART scores from the self-report questionnaires, such as the BIS total score and the FFOCI total score, and age. In the second step, we included the neuropsychological tasks as predictors: CSPAN, DSPAN, 1-back, 2-back, Go/No-Go discriminability scores, and WCST perseverative errors. Correlations between these measures can be found in the Supplementary Materials, Table S2, while the results of the regression model are presented in Table 3. In the regression analysis, we did not analyze the data of those who were excluded from even one task. Thus, a total of 83 individuals' data were analyzed (see Supplementary Materials S1.1). Overall, the first model was significant (Adjusted *R*^2^ = 0.422, *F*(3, 79) = 20.984, *p* < 0.001). BIS scores demonstrated a moderate positive relationship with WART scores (*β* = 0.393, *p* < 0.001) indicating that higher trait impulsiveness is associated with a greater risk of work addiction. In addition, FFOCI scores exhibited a strong positive relationship with WART scores (*β* = 0.608, *p* < 0.001) indicating that higher trait compulsiveness is also linked to a higher risk of work addiction. The second model showed a trend level improvement from the first model (Adjusted *R*^2^ = 0.471, *F*(9, 73) = 9.103, *p* = 0.052). Go/No-Go discriminability scores exhibited a statistically significant negative relationship with WART scores (*β* = –0.229, *p* = 0.017), suggesting that a higher risk of work addiction was associated with poorer inhibitory control. Interestingly, but consistent with the results of the ANCOVA analysis, 1-back discriminability scores had a positive effect on WART (*β* = 0.183, *p* = 0.039), indicating that higher risk of work addiction was associated with better performance in this task. Mirroring the result of the comparison between the LWA and HWA groups, age was also negatively related to WART scores (*β* = –0.228, *p* = 0.018). No other statistically significant association was observed.

**Table 3:** Unstandardized and standardized coefficients of the hierarchical multiple linear regression model, predicting WART total scores in the full sample (N = 83).

	*B*	*SE*	95% CI	*β*	*t*	*p*
Step 1: self-report questionnaires						
Intercept	−0.557	11.066	–22.584, 21.471		–0.050	0.960
BIS	0.564	0.126	0.313, 0.815	0.393	4.472	< 0.001
FFOCI	0.320	0.047	0.228, 0.413	0.608	6.883	< 0.001
Age	–0.142	0.096	–0.333, 0.049	–0.126	–1.475	0.144
Step 2: neuropsychological tasks						
Intercept	3.023	13.215	–23.313, 29.360		0.229	0.820
BIS	0.514	0.125	0.266, 0.763	0.358	4.124	< 0.001
FFOCI	0.298	0.047	0.204, 0.392	0.566	6.337	< 0.001
DSPAN	–0.380	0.813	–2.001, 1.240	–0.044	–0.468	0.641
CSPAN	1.932	1.410	–0.879, 4.742	0.142	1.370	0.175
1-back discriminability score	3.085	1.466	0.163, 6.007	0.183	2.104	0.039
2-back discriminability score	–2.319	1.702	–5.711, 1.072	–0.127	–1.363	0.177
Go/No-Go discriminability score	–3.660	1.503	–6.656, –0.664	–0.229	–2.434	0.017
CST perseverative errors	0.504	0.361	–0.216, 1.224	0.119	1.394	0.167
Age	–0.256	0.106	–0.467, –0.044	–0.228	–2.410	0.018

*LWA* low risk group for work addiction, *HWA* high risk group for work addiction, *WART* work addiction risk test, *BIS* Barratt impulsiveness scale, *FFOCI* five-factor obsessive–compulsive inventory short form, *DSPAN* digit span task, *CSPAN* counting span task, *CST* card sorting task.

We also fit the regression model in the two subgroups of participants separately. The result of this analysis can be found at Supplementary Materials, S3.

## Discussion

Individuals with work addiction experience persistent, uncontrollable thoughts about work, have difficulties distracting themselves from their work, and exhibit excessive concerns about their achievements. Several theoretical models^75–77^ and empirical studies^34,36,76^ have indicated a possible relationship between the aforementioned symptoms and cognitive dysfunctions related to this form of behavioral addiction. Therefore, our objective was to investigate executive functions, specifically inhibitory control, cognitive flexibility, and working memory, in individuals categorized as high and low risk for work addiction. The aim was to identify the specific neuropsychological functions that show alterations associated with work addiction. Additionally, this study aimed to compare trait impulsiveness and compulsiveness between the HWA and LWA groups, as these personality traits have been linked to executive functions, particularly inhibitory control, and cognitive flexibility. To the best of our knowledge, this was the first study to utilize self-report questionnaires in combination with neuropsychological reaction time tasks to assess various executive functions in work addiction.

The results of our study indicate that the HWA group exhibits deficiencies in inhibitory control and those working memory functions that are more complex and involve updating. Conversely, in the simpler 1-back task, where the focus is on maintaining a representation of presented items in memory rather than continuously updating information as in working memory, the HWA group outperformed the LWA group. In addition, according to the results of regression analysis, work addiction was predicted by higher performance on 1-back task and lower performance on the Go/No-Go task, which measures inhibitory control. At the same time, cognitive flexibility is not found to be altered in work addiction, despite the higher self-reported impulsiveness and compulsiveness among the individuals with work addiction.

We observed a distinct divergence between the risk of work addiction and scores in the 1-back and 2-back tasks: 1-back scores showed a positive association with work addiction risk, while 2-back scores were inversely related to it. One intriguing explanation for these findings is that the 1-back task primarily focuses on the maintenance and storage of information without imposing much of a working memory load, while the 2-back task demands more complex cognitive processes like updating and manipulating information^78^. It is conceivable that certain traits characterizing individuals with work addiction, such as perseverance, conscientiousness, and perfectionism^79–81^, may offer advantages in simpler tasks that require only maintenance and storage of information or sustained attention. These qualities can lead to accurate responses when the task involves only a single step back. However, in more complex tasks demanding updating, they might demonstrate poorer performance. The general overload associated with individuals affected by work addiction^82^ could influence their working memory in such situations. Additionally, the improved performance in the 1-back task raises the possibility that workaholics, who more frequently have intellectual occupations and higher education^4,5^, might exercise their brains in a way that preserves certain cognitive functions, including working memory. Alternatively, it is plausible that individuals with slightly better working memory are more susceptible to developing work addiction compared to other, more severe addictions. In contrast, conditions like drug addiction or gambling disorder are often associated with weaker working memory functions^83,84^, potentially making them less protective against the development of clinical disorders. However, it is essential to emphasize that these are speculative explanations regarding the associations, and further (mainly longitudinal) research is needed to explore whether better working memory contributes to a person's susceptibility to work addiction or vice versa.

Another important finding is that the HWA group exhibited lower reaction time for the false alarm measure in the 2-back task compared to the LWA group. In n-back tasks, the primary focus is on comparing the current stimulus with one that occurred earlier, all while filtering out any distracting stimuli. Specifically, in a 2-back task, the person must effectively hold and manage multiple stimuli simultaneously to perform the task successfully. The faster reaction time for false alarms might be because those with work addiction struggle to hold onto and pick the right information, which could make them make quick, impulsive choices. It is possible that individuals with work addiction, due to the alterations in more complex working memory tasks, are more susceptible to heightened impulsiveness when faced with decision-making situations. The decreased working memory performance observed in the 2-back task may play an important role in multitasking observed in work addiction, as previous research has established a notable association between multitasking errors and updating^85^. While multitasking in work addiction has not yet been investigated, some authors have emphasized its potential relevance in work addiction^86,87^. Moreover, the WART, designed to assess the likelihood of work addiction, includes items specifically addressing multitasking behavioral patterns^42^. Empirical studies have revealed that individuals with work addiction, despite spending more time on their work, often exhibit poorer performance compared to others^36^. This may be attributed to the utilization of inefficient working methods and strategies, such as multitasking. Considering that updating is responsible for the continuous monitoring and manipulation of information in working memory^11,88^, future studies should explore how weaker updating processes in work addiction might be associated with daily functioning, especially in terms of working strategies and overall performance in more complex situations.

Our results support our hypothesis, indicating that the HWA group exhibits lower inhibitory control compared to the LWA group. This finding implies that individuals with work addiction may face challenges in suppressing certain thoughts and impulses, potentially leading to more impulsive behaviors. In light of the central role of inhibitory control in higher executive functions, such as organization, planning, and regulation^12,89^, any decline in its performance has the potential to significantly impact the overall quality of life for these individuals. A previous study identified that individuals with work addiction tend to experience challenges in regulating their emotions and persist in their behaviors even when faced with unfavorable outcomes^79^. Moreover, these individuals tend to experience elevated levels of perceived stress, engage in rumination, and exhibit symptoms of mood disorders^64,90,91^. These emotional regulation characteristics can potentially be associated with alterations in inhibitory control. Impulsive behavior can also have an impact on the dynamics of social relationships, as it tends to exacerbate conflicts commonly encountered by individuals with work addiction, whether in familial or occupational settings^81,82^.

Our results regarding inhibitory control align with research findings that suggest a potential connection between work addiction and ADHD^86^. In ADHD, inhibition processes are often underactive, which is also associated with challenges in attention and concentration. Additionally, workaholism often correlates with individuals taking on excessive tasks, leading to feelings of overwhelm. Individuals with work addiction tend to agree to and undertake numerous tasks without careful consideration, often exceeding their capacity to manage, ultimately resulting in levels of workaholic behavior^86^. Moreover, it is worth noting that deficiencies in inhibitory control may be parallel to work addiction, as observed in other addictive disorders. This cognitive under-functioning can be observed in both substance-related (e.g., alcohol and drug addiction) and behavioral addictions (such as gambling and gaming disorder, and problematic internet use)^92–94^. Thus, in this regard, work addiction exhibits similar neuropsychological characteristics to other forms of addiction.

An intriguing finding is that, while aligning with earlier research^36,39–41^, we observed heightened levels of impulsivity and compulsivity within the HWA group. However, these personality traits did not demonstrate a correlation with the functioning of inhibitory control and false alarm reaction time. These results suggest the presence of impulsive behaviors in work addiction, yet the absence of connections between these variables implies that these types of impulsive responses may manifest differently in one’s life. To gain further insights into the potential role of altered inhibitory control in work addiction, future research should consider incorporating additional behavioral tasks and questionnaires that measure higher-level executive functions, with a particular focus on decision-making processes and emotional regulation.

Contrary to our hypothesis, our findings indicate no discernible distinction between the two groups in cognitive flexibility. Regarding this executive function, work addiction differs from other behavioral addictions, as previous studies have identified impairments in this cognitive function in gaming and gambling disorders^20,22,26,27^. However, it is important to note that although we found no difference in behavioral performance for this neurocognitive function, there may be altered functioning at the neural level. In a functional magnetic resonance imaging (fMRI) study conducted by Ding et al.^95^ with individuals with gaming disorder, no differences were observed at the behavioral level, but hyperactivation in several brain areas (e.g., medial frontal gyrus, right anterior cingulate cortex) was found in the group with addiction. This result suggests that the behavioral performance may have been compensated for at the neural level, requiring greater neural involvement to achieve similar performance. In future research, incorporating brain imaging techniques could be crucial in investigating potential differences at neural level in work addiction.

It is important to acknowledge the limitations of this study when interpreting the findings on cognitive functioning in work addiction. Similar to other addictive disorders, work addiction can coexist with psychiatric disorders such as obsessive–compulsive personality disorder, ADHD, depression, or other addictions^86,96–98^. Although individuals with these comorbidities were excluded from our study, it is possible that these psychiatric disorders could contribute to the variability of executive functioning observed in work addiction. Future studies should investigate whether our findings can be generalized to a more heterogeneous work addiction group that includes individuals with a range of comorbidities. The small sample sizes in both groups, partly due to these exclusions, could also impact the generalizability of our results. Additionally, it is important to note that some of the functions were represented by only one indicator task in this study, which can make it challenging to differentiate between function-specific effects and task-specific effects. Moreover, because of the task impurity problem, many classical neuropsychological tasks require the engagement of multiple functions for successful performance, reflecting the interdependent nature of these functions and potentially muddying the clarity of function-specific findings. This highlights the necessity for future research to employ a broader range of tasks, allowing for a clearer distinction between these effects. Given that our analysis focused primarily on relationships rather than causation, it raises questions about the involvement of working memory deficits in the development and persistence of work addiction. If working memory deficits are a precursor to work addiction, then this could potentially contribute to a variety of challenges in individuals' lives, including distractions, fatigue, and impairment in work performance. Another possibility to consider is that continuous overload, potentially resulting from work addiction, leads to a decline in working memory performance, especially in more complex tasks. However, if a general overload were the primary cause, we would expect to observe a similar impact across the other executive functions as well. Obtaining a precise answer to this question would require longitudinal or experimental research. Precisely because of this another important aspect that warrants further investigation is the phenomenon of burnout, which is commonly associated with work addiction^36^. Consequently, future research endeavors should consider including assessments of burnout and controlling for its influence during data analysis. These considerations highlight the need for future research to encompass a more diverse work addiction population, account for comorbidities, address sample size limitations, and explore the impact of burnout on cognitive functioning.

In the present study, our aim was to investigate executive functions and related personality traits in work addiction. Our results suggest a selective impairment of executive functions in work addiction. We observed weaker inhibitory control and alterations in certain working memory processes. Specifically, individuals with work addiction performed better on the simpler 1-back task that required sustained attention, but they struggled with the more complex working memory functions associated with the updating process. There were no discernible differences between the groups in regard to other working memory tasks, including digit span and counting span tasks. Therefore, it is plausible to attribute the moderate relationships typically observed in our results concerning working memory capacity to the varying cognitive demands associated with these tasks. Cognitive flexibility is not found to be altered in work addiction, despite finding higher levels of self-reported compulsiveness and impulsiveness in work addiction. Since inhibitory control and working memory play important roles in higher order executive functions^12,89^, the alteration of these functions is potentially associated with modified decision-making, working strategies, and even social functioning. It turns out that the neuropsychological profile of work addiction is partly similar and partly different from other addictive disorders. Individuals with work addiction do not exhibit difficulties in certain areas of working memory and cognitive flexibility, which is often seen in other addictions. In fact, they even outperform others up to a certain point on specific working memory tasks. This underscores the idea that individuals with work addiction are not accidentally more functional in life than those with other addictive disorders. This might be why they appear less frequently in treatment centers and their harms are less visible compared to other addictions. However, it is important to note that individuals with work addiction do display deficits in inhibitory control and more complex working memory tasks, similar to individuals with other addictions. This provides evidence supporting the characterization of work addiction as an addictive disorder.

The present study represents a pioneering endeavor in exploring the neurocognitive underpinnings of work addiction. Our findings contribute to a deeper understanding of the fundamental nature of this behavioral addiction. Furthermore, these insights, accompanied by further research, could lay the foundation for the development of diagnostic protocols and therapeutic interventions aimed at addressing and mitigating the adverse effects of work addiction.

### Supplementary Information

Supplementary Information.

## Data Availability

All data are available on the following links: https://osf.io/fj9zp/?view_only=5f400c12eb8146df84cc43590eb01531; https://github.com/vekteo/Card_sorting_jsPsych; https://github.com/vekteo/Nback_jsPsych; https://github.com/vekteo/GoNoGo_jsPsych.
