# Supplementary material for: The neuropsychological profile of work addiction
Source: Sci Rep. 2023 Nov 16;13:20090. doi: 10.1038/s41598-023-47515-9 (PMC10654659; doi:10.1038/s41598-023-47515-9)
Supplement: Supplementary file 1 — Supplementary Information. [file 41598_2023_47515_MOESM1_ESM.docx]

**Supplementary Materials**

**S1. Further methodological details**

**S1.1 Quality control of the data**

In the present study, a total of 111 individuals initially participated. However, a subset of these participants was subsequently excluded from the comprehensive analysis. Six participants had diagnosed psychiatric disorders, including depression, ADHD, autism spectrum disorder, panic disorder, or generalized anxiety disorder. An additional four participants had other addictions, specifically cannabis use and gaming disorders. These addictions were assessed using the Problematic Online Gaming Questionnaire Short Form (POGQ_SF)^1^ and the Cannabis Use Disorder Test Revised (CUDIT-R)^2^. This allowed for a comparative analysis between the high-risk work-related addiction group and these other groups in later stages of the research.

Furthermore, some participants were excluded from specific tasks for various reasons. Two participants were removed from the Card Sorting Test: one due to a misinterpretation of the task and the other due to their professional background as a psychologist, which made them aware of the task's purpose and design. In the case of both the DSPAN and CSPAN tasks, procedural errors by the investigator led to the exclusion of the results. For the N-Back tasks, one participant opted out, citing the task's high difficulty level, and for another, data saving was unsuccessful. Moreover, individuals who performed more than two standard deviations from the mean in each task were also excluded. Importantly, it should be noted that the exclusion of these individuals did not significantly impact the results of the analyses. The complete data table, including outliers, is also available on the OSF platform.

**S1.2 Comparison of sex, place of residence, educational level, and age between the groups**

**Table S1. Comparison of sex, place of residence, educational level, and age between the groups**

| Variable | Statistics  (t / Mann-  Whitney U) | *df* | *p* |
| --- | --- | --- | --- |
| Sex | 1054.500 | - | .203 |
| Place of Residence | 1405.000 | - | .074 |
| Educational Level | 1210.500 | - | .991 |
| Age | 2.711 | 97 | **.008** |

*Note.* Statistical significance at *p* < 0.05 is indicated by boldfacing.

**S2. Correlational matrix**

**Table S2. Pearson’s correlation coefficients among neurocognitive tasks and personality scales**

| Variable | WART | Age | Go/No-Go discrimi-nability score | 1-back discrimi-nability score | 2-back false alarm reaction time | 2-back discrimi-nability score | CST perseverative error | DSPAN | CSPAN | BIS | FFOCI |
| --- | --- | --- | --- | --- | --- | --- | --- | --- | --- | --- | --- |
| WART | — |  |  |  |  |  |  |  |  |  |  |
| Age | –.187 | — |  |  |  |  |  |  |  |  |  |
| Go/No-Go discriminability score | –.174 | –.247* | — |  |  |  |  |  |  |  |  |
| 1-back discriminability score | .098 | .107 | .216* | — |  |  |  |  |  |  |  |
| 2-back false alarm reaction time | –.215* | .124 | .147 | .208 | — |  |  |  |  |  |  |
| 2-back discriminability score | –.125 | .157 | .216* | .116 | .402*** | — |  |  |  |  |  |
| CST perseverative error | .058 | .066 | –.099 | –.159 | –.114 | –.172 | — |  |  |  |  |
| DSPAN | –.005 | –.053 | .230* | –.015 | .110 | .341*** | –.038 | — |  |  |  |
| CSPAN | –.067 | .147 | .253** | .004 | .115 | .461*** | –.178 | .473*** | — |  |  |
| BIS | .328*** | .014 | –.107 | –.073 | –.075 | –.048 | –.128 | –.065 | –.040 | — |  |
| FFOCI | .529*** | –.170 | –.085 | .161 | –.052 | .022 | .069 | .031 | –.054 | –.226* | — |

*Note*. * *p* < .05; ** *p* < .01; *** *p* < .001

**S2. Results of the linear regression in the HWA and LWA groups**

We were also interested in exploring whether these relationships are different within the HWA and LWA subgroups. We therefore also fit the regression model in the two subgroups of participants separately. In the LWA subgroup, the model showed an adequate, but noticeably worse fit, Adjusted *R^2^* = .095, *F*_(9, 39)_ = 1.557, *p* = .163. The effects of BIS (*β* = .272, *p* = .077) and FFOCI were observed again (*β* = .506, *p* = .002), with attenuated effect sizes and the former being reduced to trend level significance. In the HWA subgroup, the model showed an adequate, but noticeably worse fit, Adjusted *R^2^* = .153, *F*_(9, 24)_ = 1.660, *p* = .154. The effects of BIS (*β* = .436, *p* = .038) and FFOCI were observed again (*β* = .443, *p* = .046). There were effects of 1-back discriminability score (*β* = .375, *p* = .077) and 2-back discriminability score (*β* = –.414, *p* = .050) in opposite directions, although the former effect did not reach statistical significance in this group.

Overall, the regression analyses suggest that there is a strong positive relationship between trait impulsiveness and compulsiveness and work addiction risk, that is consistent across the entire range of the work addiction risk spectrum. They also suggest that there is a tentative relationship between work addiction risk and working memory, as measured by the 1-back and 2-back tasks, and inhibitory control, as measured by the Go/No-Go task. Higher work addiction risk seems to be associated with weaker working memory performance and inhibitory control. However, there is also a surprising dissociation between the associations of work addiction risk with 1-back and 2-back scores, as 1-back scores were positively, and 2-back scores were negatively related to work addiction risk, especially among high work addiction risk participants

**Table S3. Unstandardized and standardized coefficients of the multiple linear regression model, predicting WART total scores in the LWA subgroup (N = 49)**

|  | ***B*** | ***SE B*** | **95% *CI*** | ***β*** | ***t*** | ***p*** |
| --- | --- | --- | --- | --- | --- | --- |
| **Intercept** | 19.466 | 14.213 | –9.282, 48.214 |  | 1.370 | .179 |
| **BIS** | 0.271 | 0.149 | –0.030, 0.573 | 0.272 | 1.819 | .077 |
| **FFOCI** | 0.182 | 0.056 | 0.069, 0.295 | 0.506 | 3.271 | .002 |
| **DSPAN** | 0.442 | 0.846 | –1.268, 2.152 | 0.086 | 0.523 | .604 |
| **CSPAN** | 0.441 | 1.459 | –2.511, 3.393 | 0.053 | 0.302 | .764 |
| **1-Back discriminability score** | 0.406 | 1.641 | –2.914, 3.725 | 0.037 | 0.247 | .806 |
| **2-Back discriminability score** | –1.084 | 1.791 | –4.706, 2.538 | –0.094 | –0.605 | .549 |
| **Go/No-go discriminability score** | –1.015 | 1.609 | –4.269, 2.240 | –0.105 | –0.631 | .532 |
| **CST perseverative errors** | –0.142 | 0.442 | –1.037, 0.753 | –0.047 | –0.321 | .750 |
| **Age** | 0.008 | 0.113 | –0.220, 0.235 | 0.012 | 0.068 | .946 |

*Note*. LWA = Low risk group for work addiction; WART = Work Addiction Risk Test; BIS = Barratt Impulsiveness Scale; Five-Factor Obsessive-Compulsive Inventory Short Form (FFOCI); DSPAN = Digit Span Task, CSPAN = Counting Span Task, CST = Card Sorting Task

**Table S4. Unstandardized and standardized coefficients of the multiple linear regression model, predicting WART total scores in the HWA subgroup (N = 34)**

|  | ***B*** | ***SE B*** | **95% *CI*** | ***β*** | ***t*** | ***p*** |
| --- | --- | --- | --- | --- | --- | --- |
| **Intercept** | 16.108 | 18.436 | –21.943, 54.159 |  | 0.874 | .391 |
| **BIS** | 0.337 | 0.154 | 0.021, 0.654 | 0.436 | 2.198 | .038 |
| **FFOCI** | 0.147 | 0.070 | 0.003, 0.292 | 0.443 | 2.104 | .046 |
| **DSPAN** | 0.925 | 1.139 | –1.426, 3.276 | 0.153 | 0.812 | .425 |
| **CSPAN** | 2.598 | 1.916 | –1.356, 6.552 | 0.292 | 1.356 | .188 |
| **1-Back discriminability score** | 3.546 | 1.918 | –0.413, 7.504 | 0.375 | 1.849 | .077 |
| **2-Back discriminability score** | –4.461 | 2.166 | –8.932, 0.010 | –0.414 | –2.059 | .050 |
| **Go/No-go discriminability score** | –0.742 | 2.272 | –5.431, 3.947 | –0.070 | –0.326 | .747 |
| **CST perseverative errors** | 0.452 | 0.443 | –0.463, 1.367 | 0.209 | 1.020 | .318 |
| **Age** | 0.016 | 0.169 | –0.333, 0.366 | 0.018 | 0.097 | .923 |

*Note*. HWA = High risk group for work addiction; WART = Work Addiction Risk Test; BIS = Barratt Impulsiveness Scale; Five-Factor Obsessive-Compulsive Inventory Short Form (FFOCI); DSPAN = Digit Span Task, CSPAN = Counting Span Task, CST = Card Sorting Task **Figure S1.** WART total scores and 2-back discriminability score, BIS and FFOCI scores, separately in the LWA and HWA groups.


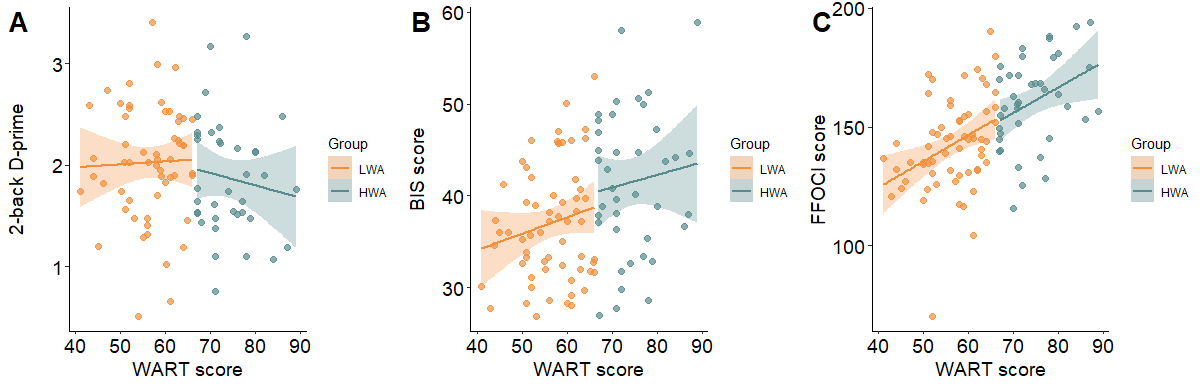


*Note*. Individual data points are participants, lines and shaded areas are least squares regression estimates and their 95% CI, respectively.

**S3. Comparison between the groups without controlling for age**

Statistical analysis

We compared the performance on tasks and questionnaire scores of the LWA and the HWA groups of work addiction. Normality was tested using the Shapiro-Wilk test. If the normality assumption was violated, Mann-Whitney U test was performed. If not, Levene’s test was applied to assess the homogeneity of variances. Welch's adjusted t-statistic was employed if Levene's test revealed a significant variance difference; otherwise, an independent sample t-test was applied. We calculated effect size using Cohen’s d measure.

**Results**

To investigate the association between work addiction and working memory performance, we compared the performance of the HWA and LWA groups on the N-back, CSPAN, and DSPAN scores. We did not find a significant difference between HWA and LWA groups on the scores of the 1-back task. However, the HWA group had a significantly lower hit score on the 2-back test (*t(92)* = 2.180, *p* = 0.032, *M*_LWA_= 13.737, *SD*_LWA_= 2.813*,* *M*_HWA_= 12.351, *SD*_HWA_= 3.293*,* *d’* = 0.453), and they were significantly faster for false alarms than the LWA group (*t(89)* = 3.188, *p* = 0.002, *M*_LWA_= 811.456, *SD*_LWA_= 157.839, *M*_HWA_= 708.735, *SD*_HWA_= 131.734, *d’* = 0.707). There was no significant difference on the other scores of this task (see Table S5).

**Table S5. The results of the independent sample T-tests and Mann-Whitney tests**

| **Variable**  **N**  **(HWA, LWA)** | **HWA** | | | **LWA** | | | | **Statistics** | | | | |  |
| --- | --- | --- | --- | --- | --- | --- | --- | --- | --- | --- | --- | --- | --- |
|  | *M* | *Med* | *SD* | | *M* | *Med* | *SD* | *df* | *t* | *U* | *p* | Cohen’s *d* | Cohen’s *d* 95% CI |
| **Go/No-Go**  98  (38, 60) |  |  |  | |  |  |  |  |  |  |  |  |  |
| Number of hits | 228.816 | 230 | 16.116 | | 233.583 | 235 | 15.503 | – | – | 920.5 | 0.109 | 0.301 | [–0.109, 0.710] |
| Number of false alarms | 27.053 | 27.5 | 9.639 | | 24.850 | 22.5 | 12.211 | 96 | –0.941 | – | 0.349 | 0.200 | [–0.607, 0.208] |
| Discriminability score | 1.542 | 1.505 | 0.563 | | 1.793 | 1.732 | 0.719 | 91.638 | 1.925 | – | 0.057 | 0.389 | [–0.023, 0.797] |
| **CST**  99  (39, 60) | 7.154 | 7 | 2.691 | | 6.650 | 6 | 2.057 | – | – | 1284 | 0.408 | 0.210 | [–0.607, 0.202] |
| **1-back**  92  (35, 57) |  |  |  | |  |  |  |  |  |  |  |  |  |
| Number of hits | 16.143 | 17 | 2.952 | | 17 | 17 | 2.486 | – | – | 847 | 0.222 | 0.314 | [–0.112, 0.737] |
| Number of false alarms | 1.4 | 1 | 1.850 | | 1.298 | 1 | 1.762 | – | – | 1029 | 0.790 | 0.056 | [–0.477, 0.365] |
| Reaction time of hits | 546.857 | 536 | 70.271 | | 562.807 | 551 | 89.447 | 90 | 0.898 | – | 0.372 | 0.198 | [–0.224, 0.620] |
| Reaction time of false alarms  57  (24, 33) | 612.7 | 605 | 108.828 | | 574.485 | 547 | 152.23 | – | – | 413.5 | 0.125 | 0.289 | [–0.846, 0.271] |
| Discriminability score | 3.116 | 3.046 | 0.698 | | 3.033 | 3.062 | 0.638 | 90 | –0.586 | – | 0.559 | 0.124 | [–0.546, 0.297] |
| **2-back**  94  (37,57) |  |  |  | |  |  |  |  |  |  |  |  |  |
| Number of hits | 12.351 | 13 | 3.293 | | 13.737 | 14 | 2.813 | 92 | 2.180 | – | 0.032 | 0.453 | [0.030, 0.871] |
| Number of false alarms | 5.649 | 5 | 3.490 | | 6.140 | 5 | 3.971 |  | – | 1029.5 | 0.846 | 0.131 | [–0.283, 0.545] |
| Discriminability score | 1.871 | 1.763 | 0.568 | | 2.025 | 2.031 | 0.566 | 92 | 1.289 | – | 0.201 | 0.272 | [–0.172, 0.660] |
| Reaction time of hits | 704.432 | 701 | 134.087 | | 734.596 | 743 | 111.551 |  | – | 918 | 0.291 | 0.245 | [0.266, 1.143] |
| Reaction time of false alarms  91  (34, 57) | 708.735 | 709 | 131.734 | | 811.456 | 791 | 157.839 | 89 | 3.188 | – | 0.002 | 0.707 | [–0.145, 0.687] |
| **DSPAN**  100  (39, 61) | 6.54 | 7 | 0.996 | | 6.92 | 7 | 1.32 | – | – | 950,5 | 0.082 | 0.325 | [–0.081, 0.728] |
| **CSPAN**  100  (39, 61) | 3.376 | 3.333 | 0.718 | | 3.661 | 3.667 | 0.813 | 98 | 1.788 | – | 0.077 | 0.372 | [–0.035, 0.776] |
| **BIS**  101  (39, 62) | 41.462 | 41 | 7.853 | | 37 | 36 | 6.317 | – | – | 1615 | 0.005 | 0.626 | [–1.038, –0.210] |
| **FFOCI**  101  (39, 62) | 160.538 | 160 | 18.982 | | 142.661 | 142.5 | 19.666 | 99 | –4.507 | – | < 0.001 | 0.925 | [–1.347, –0.498] |

*Note*. LWA = Low risk group for work addiction; HWA = High risk group for work addiction; WART = Work Addiction Risk Test; BIS = Barratt Impulsiveness Scale; Five-Factor Obsessive-Compulsive Inventory Short Form (FFOCI); DSPAN = Digit Span Task, CSPAN = Counting Span Task, CST = Card Sorting Task

Even though the HWA group showed a weaker performance on the DSPAN (*U* = 950.5, *p* = 0.082, *Md*_LWA_= 7, *Md*_HWA_= 7, *d’* = 0.325) and CSPAN tasks (*t(98)* = 1.788, *p* = .077, *M*_LWA_= 3.661, *SD*_LWA_= 0.813, *M*_HWA_= 3.376, *SD*_HWA_= 0.718, *d’* = 0.372), the differences between the groups were only approaching significance.

To explore the possible difference between the LWA and HWA groups in the performance of the CST measuring cognitive flexibility, Mann-Whitney test was performed. The results did not show a significant difference between the LWA and HWA groups for the average of perseverative errors in the CST (*U* = 1284, *p* = .408, *Md*_LWA_= 6 *Md*_HWA_= 7, *d’* = 0.210).

According to the results of the Mann-Whitney test, there was no significant group difference on the hit score of the Go/no-Go tasks (*U* = 920.5, *p* = .109, *Md*_LWA_= 235, *Md*_HWA_= 230, *d’* = 0.302). The independent sample t test did not show a significant difference on the false alarm scores (*t*(96) = –0.941, *p* = .349, *M*_LWA_= 24.850, M_HWA_= 27.053, *d’* = 0.200) either (Table S5). The Welch's t-test showed a nonsignificant, trend-level group difference in the discriminability score, as the HWA group scored lower than the LWA group (*t*(91.638) = 1.925, *p* = .057, *M*_LWA_ = 1.793, *M*_HWA_ = 1.542, *d’* = 0.388).

The results of the independent sample t-test revealed a statistically significant difference in FFOCI scores, with the HWA group obtaining higher scores than the LWA group (*t(99)* = –4.507, *p <* .001, *M*_LWA_ = 142.661, *M*_HWA_ = 160.538, *d’* = 0.925). Likewise, the HWA group scored significantly higher on the BIS than the LWA group (*U* = 1615, *p* = .005, *Md*_LWA_= 36, *Md*_HWA_= 41, *d’* = 0.626).

**S4. Results of the robust ANCOVA**

For Go/No-Go hits, the Shapiro-Wilk test indicated non-normality in both the HWA (*p* = .037) and the LWA groups (*p* < .001). In the robust ANCOVA, significant group differences emerged at ages 28 (*p* = .023), and 35 (*p* = .044), and trend level differences at ages 31 (*p* = .050) and 40 (*p* = .072), with a smaller number of hits in the HWA group in each case.

For the number of false alarms in the Go/No-Go task, Levene’s test indicated violation of homogeneity (*F*_(1,94)_ = 4.596, *p* < .035). In a robust ANCOVA, no significant group differences emerged at any of the tested levels of the covariate.

For DSPAN, normality was violated according to the results of the Shapiro-Wilk test, in both of the HWA (*p* = .003) and LWA (*p* < .001) groups. In the robust ANCOVA, significant group differences emerged at age 32 (*p* = .025), with a smaller score in the HWA group compared to the LWA group.

The Shapiro-Wilk test showed that the normality of the BIS impulsiveness scores were violated too in the LWA (*p* = .025) groups. In the robust ANCOVA, significant group differences emerged at age 27 (*p* = .029) with a lower score in the HWA group compared to the LWA group.

For the number of hits and false alarms, false alarm reaction time in the 1-back task, number of false alarms in the 2-back task, number of perseverative errors in CST and the Shapiro-Wilk test showed the violation of the normality. In the robust ANCOVA, no significant group differences emerged at any age level on these scores (see Table S6).

**Table S6. Robust ANCOVA results**

| Variable | Level of age covariate | LWA-HWA difference | LWA-HWA difference SE | Test statistic | *p* |
| --- | --- | --- | --- | --- | --- |
| Go/No-Go number of hits | 28 | 11.07 | 4.49 | 2.47 | .023 |
|  | 31 | 9.02 | 4.39 | 2.05 | .050 |
|  | 35 | 7.31 | 3.47 | 2.10 | .044 |
|  | 40 | 13.63 | 6.78 | 2.01 | .072 |
|  | 43 | 6.64 | 4.15 | 1.60 | .133 |
| Go/No-Go number of false alarms | 28 | –4.69 | 3.31 | 1.42 | .172 |
|  | 31 | –4.55 | 3.15 | 1.44 | .160 |
|  | 35 | 0.47 | 3.01 | 0.16 | .876 |
|  | 40 | –.52 | 2.89 | 0.18 | .859 |
|  | 43 | –.34 | 3.53 | 0.38 | .709 |
| 1-back number of hits | 28 | 0.11 | 0.89 | 0.13 | .901 |
|  | 31 | 0.65 | 0.70 | 0.93 | .359 |
|  | 34 | 0.99 | 1.00 | 0.99 | .334 |
|  | 38 | 1.67 | 1.17 | 1.43 | .178 |
|  | 42 | 0.17 | 1.18 | 0.15 | .886 |
| 1-back number of false alarms | 28 | 0.78 | 0.48 | 1.61 | .127 |
|  | 31 | 0.85 | 0.50 | 1.71 | .100 |
|  | 34 | 0.45 | 0.43 | 1.05 | .301 |
|  | 38 | 0.28 | 0.39 | 0.70 | .490 |
|  | 42 | -0.26 | 0.48 | 0.54 | .604 |
| 1-back reaction time of false alarms | 31 | –61.32 | 38.36 | 1.60 | .135 |
|  | 32 | –61.32 | 38.36 | 1.60 | .135 |
|  | 33 | –59.33 | 37.55 | 1.58 | .141 |
|  | 34 | –59.33 | 37.55 | 1.58 | .141 |
|  | 35 | –59.33 | 37.55 | 1.58 | .141 |
| 2-back number of false alarms | 28 | 1.37 | 1.40 | 0.98 | .341 |
|  | 31 | 0.90 | 1.16 | 0.78 | .445 |
|  | 35 | 0.42 | 1.03 | 0.40 | .689 |
|  | 40 | -0.09 | 1.06 | 0.08 | .937 |
|  | 43 | 0.11 | 1.08 | 0.10 | .919 |
| CST | 28 | –0.11 | 0.79 | 0.14 | .886 |
|  | 32 | –0.34 | 0.73 | 0.47 | .642 |
|  | 38 | –0.20 | 0.55 | 0.36 | .719 |
|  | 43 | –0.72 | 0.83 | 0.87 | .401 |
|  | 44 | –0.46 | 0.86 | 0.54 | .600 |
| BIS | 27 | –6.31 | 2.64 | 2.39 | .029 |
|  | 32 | –5.17 | 2.53 | 2.04 | .052 |
|  | 38 | –4.19 | 2.48 | 1.69 | .106 |
|  | 43 | –3.56 | 2.78 | 1.28 | .227 |
|  | 44 | –2.97 | 2.61 | 1.14 | .275 |
| DSPAN | 28 | 11.07 | 4.49 | 2.47 | .023 |
|  | 31 | 9.02 | 4.39 | 2.05 | .050 |
|  | 35 | 7.31 | 3.47 | 2.10 | .044 |
|  | 40 | 13.63 | 6.78 | 2.01 | .072 |
|  | 43 | 6.64 | 4.15 | 1.60 | .133 |

*Note*. LWA = Low risk group for work addiction; HWA = High risk group for work addiction; BIS = Barratt Impulsiveness Scale; Five–Factor Obsessive–Compulsive Inventory Short Form (FFOCI); DSPAN = Digit Span Task, CSPAN = Counting Span Task, CST = Card Sorting Task

**References**

1. Pápay, O., Urbán, R., Griffiths, M. D., Nagygyörgy, K., Farkas, J., Kökönyei, G., Felvinczi, K., Oláh, A., Elekes, Z., & Demetrovics, Z. (2013). Psychometric properties of the problematic online gaming questionnaire short-form and prevalence of problematic online gaming in a national sample of adolescents. *Cyberpsychology, behavior and social networking*, *16*(5), 340–348. <https://doi.org/10.1089/cyber.2012.0484>

2. Adamson, S. J., Kay-Lambkin, F. J., Baker, A. L., Lewin, T. J., Thornton, L., Kelly, B. J., & Sellman, J. D. (2010). An improved brief measure of cannabis misuse: the Cannabis Use Disorders Identification Test-Revised (CUDIT-R). *Drug and alcohol dependence*, *110*(1-2), 137–143. <https://doi.org/10.1016/j.drugalcdep.2010.02.017>
